# Infant food users' perceptions of safety: A web-based analysis approach

**DOI:** 10.3389/frai.2023.1080950

**Published:** 2023-02-17

**Authors:** Sherman Aline, Gilles Hubert, Yoann Pitarch, Rallou Thomopoulos

**Affiliations:** ^1^Centre National de la Recherche Scientifique (CNRS), Institut de Recherche en Informatique de Toulouse (IRIT), Institut National Polytechnique (INP), University of Toulouse, Toulouse, France; ^2^Ingénierie des Agropolymères et Technologies Emergentes (IATE), Institut National de Recherche pour l'Agriculture, l'Alimentation et l'Environnement (INRAE), Institut Agro, University of Montpellier, Montpellier, France

**Keywords:** web mining, opinion, consumer, contaminants, sentiment analysis, subjectivity, modality

## Abstract

This paper aims to explore consumer beliefs about health hazards in infant foods by analyzing data gathered from the web, focusing on forums for parents in the UK. After selecting a subset of posts and classifying them by topic, according to the food product discussed and the health hazard discussed, two types of analyses were performed. Pearson correlation of term-occurrences highlighted what hazard-product pairs are most prevalent. Ordinary Least Squares (OLS) regression performed on sentiment measures generated from the texts provided significant results indicating positive or negative sentiment, objective or subjective language, and confident or unconfident modality associated with different food products and health hazards. The results allow comparison between perceptions obtained in different countries in Europe and may lead to recommendations concerning information and communication priorities.

## 1. Introduction

Essentially present in food sciences via sensory analysis and in marketing work, consumers play a particular role for several reasons: their number, their heterogeneity, the impact of their choices on the agri-food sectors and on the society, the non-coordination of their decisions, the complexity of their choices (willingness to pay, sensory preferences, awareness of nutritional and sanitary health issues, commitment to environmental and social plans, etc.), the difficulty of understanding the levers determining these choices (media, social network, etc.). In this context, the digital revolution can make it possible to increase the potential for expression of consumers, in particular in research projects. Despite noticeable attempts such as Kim and Jeong (2015), Vidal et al. (2015), and Tao et al. (2020) in different geographic areas, this theme is little represented in the panorama of existing food models, in particular at the European level (Aceves Lara et al., 2018; Djekic et al., 2019; Thomopoulos et al., 2019; Kansou et al., 2022). In this paper, we consider web mining to collect and analyze information available in the form of free declaratives (blogs, product comments, forums, etc.) as a means of reaching larger panels than commonly practiced by classic methods in the food industry. In this sense, the use of automated or semi-automated methods may bring added value compared to the knowledge obtained by traditional manual approaches for information collection.

The aim of this paper is to understand the concerns related to health hazard in infant food products, expressed online by parents. Indeed, the vulnerable public of young children under the age of three has been the object of large-scale studies carried out by health authorities to identify the main hazards in infant food (ANSES, 2016; Hulin et al., 2019). Moreover, the current European legislative framework that governs the safety of infant food sets strict requirements for their composition and labeling (European Commission, 2021). Nevertheless, improving food safety necessitates the cooperation of a wide range of stakeholders, including the household level. The results of a recent study (Franc-Dabrowska et al., 2021) highlight that public awareness is needed, and food chain stakeholders need to be trained and organized to contribute to the collective effort required to improve food safety from farm to fork. This is why in the present paper, the focus is on perceptions of infant food safety for different food products at the level of infant food users and primarily parents.

The questions addressed by the paper can be expressed as follows: Do the discussions hold by parents on web forums express concerns regarding health hazards in infant food products? For what hazards and what products more specifically? How can these concerns be characterized using sentiment analysis tools?

The methods used are presented in section **??**: these include data collection (2.1), data cleaning and selection (2.2), data classification (2.3), sentiment metrics used for the analysis (2.4), and correlation and regression methods used for the analysis (2.5). Results are presented in Section 3. Discussion and comparison with previous works is provided in Section 4, before concluding in Section 5.

## 2. Methods

**Figure 1:**
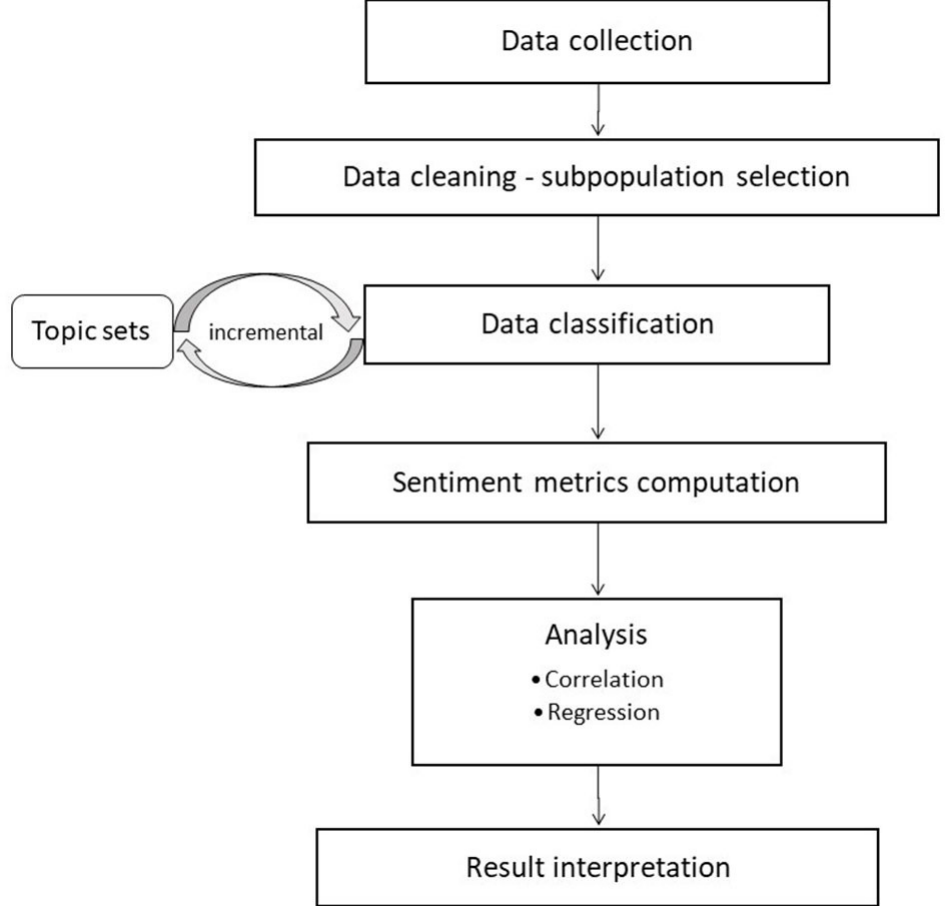
Flowchart of the method followed.

This section describes the methodological steps followed. These are displayed in the flowchart of Figure 1.

### 2.1. Data collection

To be considered relevant for the study, a source must fulfill the preliminary requirement of providing discussion about health hazards in infant foods. The food products considered were limited to four categories, following the choice of the ongoing research program “Safe food for infants” (SAFFI Project, 2020–2024): baby jars with vegetable and fish, powdered infant formula, fruit compote, and infant cereals. A list of 27 keywords composing the set of hazard topics was also pre-established by experts to search for mentions about microbiological and chemical hazards.

After considering a variety of sources, the internet forum Netmums (Netmums, 2021), located in the United Kingdom (UK), was chosen to collect relevant data. By data, here we mean a set of threads, i.e., strings of messages or “posts” that make up a conversation on a certain topic. The reasons for focusing on the UK were two-fold: on the one hand, various tools for natural language processing are operational for the English language; on the other hand, limiting the study to the European area allows the results to be compared with the conclusions of the SAFFI European project. Criteria for choosing this source include:

- Our topic is well-represented through *hazards* × *products* searches;
- It has status of reference website;
- It is easily found through different web searches (thus well web-referenced);
- UK population is well-represented;
- It performs well for commonly used indicators such as number of posts and last update.

### 2.2. Data cleaning and subpopulation selection

In order to further filter the data which are relevant to the analysis, the following criteria were used to extract a relevant and useful subsample of threads:

- Time: the thread contains posts from 2016 or later. This criterion ensures the discussion is relatively recent (no more than five years old at the date of the study);
- Minimum number of occurrences: a hazard and a product occur at least once in the thread. This criterion ensures that the thread is in the scope of the study, i.e., it contains information which can be categorized in a hazard topic and a product topic;
- Term distance: the number of words between product and hazard in the thread is below the 95th percentile. The assumption that when words are closer together, they are more likely to be related, is quite common in Natural Language Processing (Mikolov et al., 2013a,b). In this study, if hazard and product are not syntactically close together, this indicates that the product and hazard are not discussed in relation to each other.

### 2.3. Classification

After selection of a relevant subsample, topic classification is performed. Each post is assigned a topic from two sets: products and hazards. Classification is done based on the most frequently occurring terms in the post. It led to 640 classified posts. This approach is highly accurate since product and hazard categories are very specific, but the counterpart is that many posts remain unclassified (NA) due to the absence of occurrence of a product or a hazard from the predefined sets. In order to keep accuracy in exploring the data, additional product and hazard categories were created. While the initial sets of topics (products and hazards) were firstly expert-provided, the alignment of new labels allowing one to classify NA posts was supervised.

Concerning infant food products, two observations led us to dynamically enrich the product set. Firstly, specific baby food brands are sometimes mentioned, which indicates that a baby food is being discussed, but it is not feasible to classify it in the predefined categories, for instance as vegetable baby jar or as fruit puree. This explains the introduction of the categories “*baby food”* and “*jarred food”*. Secondly, posts may discuss a type of baby food without explicitly stating that it is baby food: it may be obvious by the context of the thread and detected this way. This is why two additional categories were added, “*fruit in baby context”* and “*veg in baby context”*. Both categories refer to occurrences mentioning a fruit or vegetable respectively, alongside mention of either an infant or a brand name baby food.

Concerning hazards, an additional category was created for other possible terms than those initially established, which are less specific than current SAFFI hazard interests but could be considered. The terms associated with this new category “*related terms”* are the following: *carcinogen, chemicals, toxic, toxin, poisonous, fungus, food poisoning, hazard, EFSA, European Food Safety Authority*.

### 2.4. Metrics used

Two tools were used to compute sentiment metrics: the VADER package (Hutto and Gilbert, 2014) of the Natural Language Toolkit (NLTK) (Bird et al., 2009), and the PATTERN python module (De Smedt and Daelemans, 2012). Both use a lexical approach, i.e., based on a large dictionary of terms associated with scores. NLTK has been trained on social media text, so it is ideal for our dataset. PATTERN is trained on official texts, but is also used for its unique ‘subjectivity’ and ‘modality’ metrics. The 3.6 versions of Python, VADER and PATTERN were used.

The *sentiment* metrics (computed with NLTK or PATTERN) is a real number taking their values in the interval [−1, 1]. It measures to what extent the post expresses a positive or negative sentiment, from fully negative (-1) to fully positive (1).

The *subjectivity* metrics (computed with PATTERN) is a real number in [0, 1]. It measures to what extent the post uses subjective language, from fully objective (0) to fully subjective (1).

The *modality* metrics (computed with PATTERN) is a real number in [−1, 1]. It measures to what extent the author uses a confident or credulous tone, from fully credulous (-1) to fully confident (1).

The three metrics are independent from each other (see Wankhade et al., 2022 for more details).

### 2.5. Analysis methods

Two different methods were used.

Co-occurrence counts were calculated to determine how hazard categories are distributed across different product categories within individual posts. Correlations were calculated to determine which product-hazard pairs are most prevalent in discussion, using the Pearson correlation coefficient (Stigler, 1989), also simply called *r*. To construct a list of the most correlated products and hazards, a cut-off was defined at the 5% level of significance and correlation coefficient magnitude above 0.1.

Resulting coefficients were filtered to show only statistically significant results at the 5% level, then further filtered to those with a correlation coefficient with magnitude above 0.1. The closer the coefficient is to 1, the closer the two variables are to a perfect linear relationship.

Regressions were performed on the term-counts, which can strengthen the indication of a relationship between the metrics used and a topic. The specification for the regression is OLS (Ordinary Least Squares) (Craven and Islam, 2011), chosen for its interpretability. The dependent variable is one of the metrics (*sentiment, subjectivity*, or *modality*). Independent variables are term-counts for each hazard and product topic. All different hazards and product topics are separately counted and used as different variables in the regression. The regression model considers the metrics separately because the relationship of each metrics with each topic is to be interpreted by its own (see Section 3.3).

## 3. Results

### 3.1. Classification results

**Figure 2:**
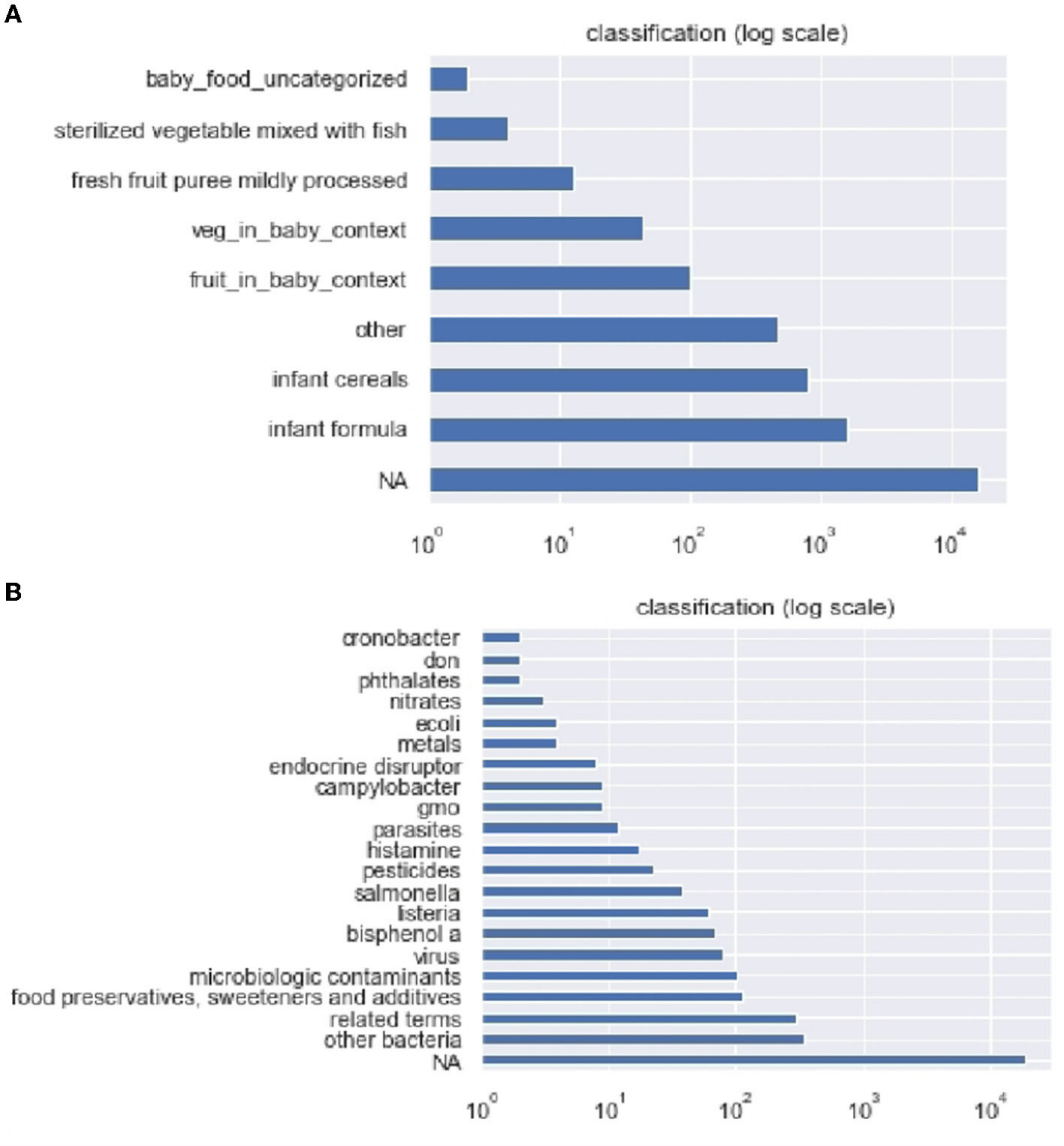
Number of posts obtained in each category **(A)** for food products and **(B)** for hazards.

Figure 2 shows the results obtained at the classification step, where each post is associated with a product category and a hazard category. The number of posts classified in each category is depicted in Figure 2A for food products and Figure 2B for hazards.

The results show that, quantitatively, the foods that appear most discussed are, by decreasing order: infant formula, infant cereals, fruit baby foods, and vegetable baby foods.

The hazards that appear most discussed are, by decreasing order: the general category of bacteria –which represents the main part of microbiological hazards–, the general category “related terms” (see Section 2.3) –which represents both chemical and microbiological hazards–, the category “food preservatives, sweeteners and additives” –which is part of the chemical hazards–, microbiologic contaminants and virus –both microbiological hazards–, then more specific hazards such as bisphenol A –chemical hazard–, *Listeria* and *Salmonella* –microbiological hazards–, etc.

### 3.2. Correlation results

**Figure 3:**
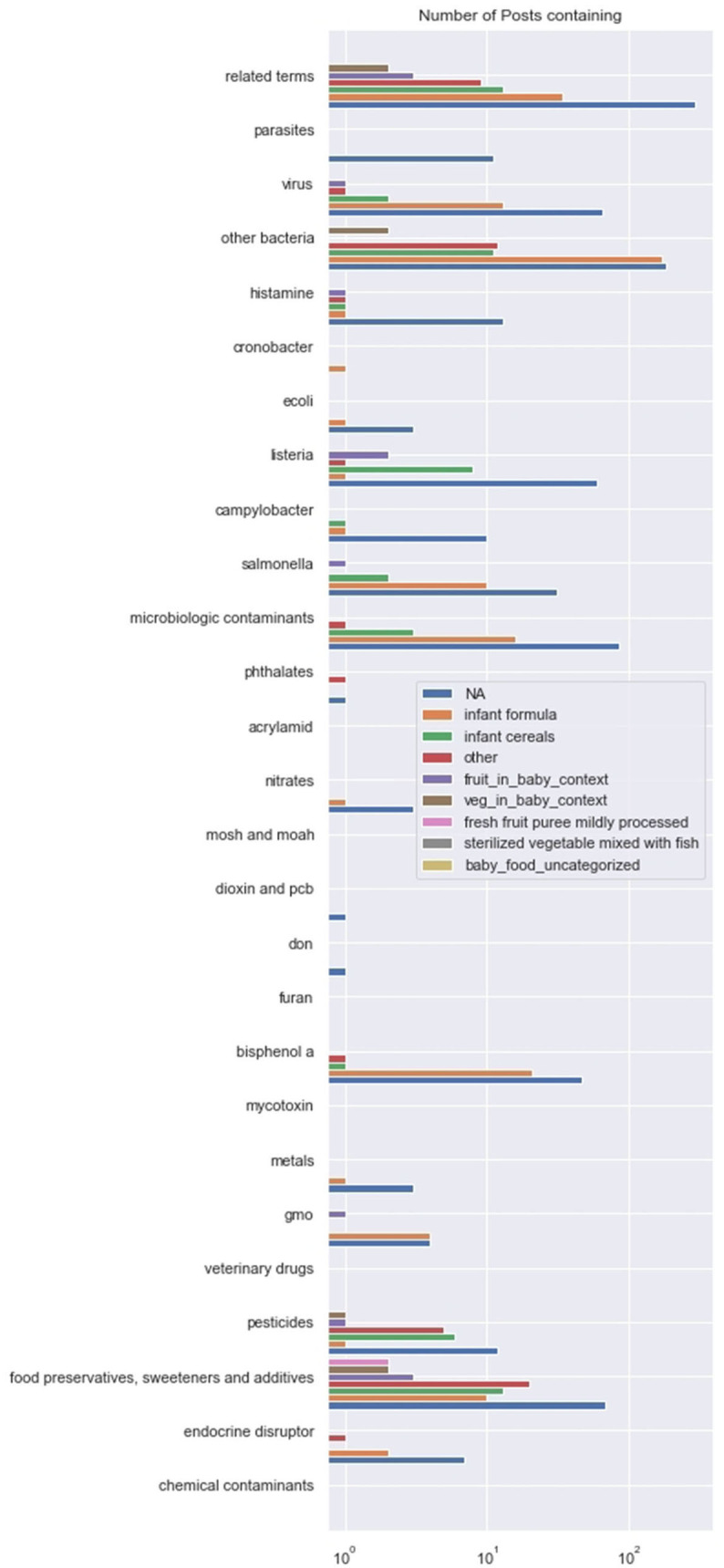
Product-hazard co-occurrences. The pairs (product, hazard) correlated at the 5% level, and their correlations, are: (jarred foods, microbes, 0.35), (formula, bacteria, 0.26), (jarred foods, pesticides, 0.16), (baby foods, preservatives, 0.11).

The plot of Figure 3 shows the number of posts containing terms from each hazard category, with a legend for each product category. This allows us to compare if some hazards are more mentioned for a specific product while also comparing the hazards among themselves. The x-axis, count of posts, is on log scale which helps us to compare levels in low-count categories.

Bisphenol A is very prevalent among discussion of infant formula, but not other products. Preservatives, sweeteners, and additives are prevalent among all product categories and particularly infant cereals. Pesticides are prevalent among discussions of infant cereals. Microbiological hazards are prevalent among the infant formula and infant cereal categories.

From the product-hazard correlations computed, the following pairs were significant at the 5% level: (jarred food, microbes), (formula, bacteria), (jarred food, pesticides), (baby food, preservatives).

### 3.3. Regression results

Table 1 sums up the regression results obtained, indicating the hazard and food categories above (+) or below (-) the baseline (NA), regarding their relationships with NLTK sentiment metrics (Table 1A), PATTERN sentiment metrics (Table 1B), subjectivity metrics (Table 1C), and modality metrics (Table 1D). Significance is at the 5% level. Detailed result tables are provided in Aline (2021).

**Table 1:** Regression result summary.

	**Hazards**	**Products**
**(A)**		
**NLTK sentiment significant results**		
+	Bisphenol a	infant cereals
−	Related terms, listeria, campylobacter	
**(B)**		
**Pattern sentiment significant results**		
+	Bisphenol a, campylobacter	infant formula
−	Endocrine disruptor	
**(C)**		
**Subjectivity significant results**		
+	Bisphenol a	
−	Campylobacter, related terms	
**(D)**		
**Modality significant results**		
+	Bisphenol a	
−	Cronobacter	

With NLTK sentiment we find that related terms, *Listeria*, and *Campylobacter* have significant negative relationships to sentiment. That is, the more these terms are used, the more negative a post is likely to be. Significant results with a positive coefficient –which is rather unexpected and further discussed in section 4– are obtained for bisphenol A, and, within the food products, for infant cereals. PATTERN sentiment agrees with this result for bisphenol A, but contradicts it for *Campylobacter*. It also identifies negative sentiment for endocrine disruptor, and positive sentiment for infant formula.

In subjectivity, bisphenol A also has a positive coefficient. *Campylobacter* and related terms and have negative coefficients. This means that the more bisphenol A is mentioned in a post, the more subjective the post is. The opposite is true for *Campylobacter* and related terms.

In modality, bisphenol A is found to have a positive coefficient and *Cronobacter* a negative coefficient. This means the more bisphenol A is mentioned, the more likely a post is to express confidence in the topic. The opposite stands for *Cronobacter*, expressing credulousness in the topic.

## 4. Discussion

In this section, the results obtained in this study from forums for parents in the UK, are compared with the conclusions of previous research papers, obtained in other European countries.

Overall, the results obtained in this study demonstrate that the largest amount of discussion of hazardous products is involved in baby formula. This confirms the results recently obtained in France by Kurtz and Thomopoulos (2021b) through a survey on 1,750 people representative of the general population. Priority concerns about infant formula thus seem common to several European countries. Nevertheless, the rest of the ranking of food products raising safety concerns among consumers seems to differ in UK and France. Indeed, for infant cereals, the results are opposite between UK and France: concern is expressed in the present UK results, whereas the absence of concern is prevalent in the French results of (Kurtz and Thomopoulos, 2021b). This may be explained by different consumption habits, since infant cereals is clearly a less widely used product than infant formula in France (Kurtz and Thomopoulos, 2021a).

Concerning the ranking of the hazards, the results obtained in this study show very balanced concerns between microbiological (categories ranked 1 and 4 are microbiological) and chemical hazards (categories ranked 2 and 3 are chemical). Previous results in France demonstrated a significant prevalence of chemical hazards, both in the survey approach of Kurtz and Thomopoulos (2021b) and on the Web (Sandjong-Sayon et al., 2022), where the hazard priority ranking identified was: heavy metals, dioxins, furan, ahead of microbiological categories. Again possible differences between both countries are highlighted through the present study.

The regression results obtained with regard to bisphenol A indicate positive sentiments in both methods used (NLTK and PATTERN), which may seem surprising since positive sentiment tends to indicate parents are not worried but feel safe. This can be explained by most discussion of bisphenol A being in the context of bisphenol A-free plastics. We could verify this hypothesis using counts of off-topic correlations, where “free” was correlated with bisphenol A. In addition, since bisphenol A co-occurs with infant formula in discussion (see section 3.2), this observation also explains why infant formula was found to be associated with positive sentiment in the PATTERN method. Indeed, posts dealing with infant formula often actually deal with bisphenol A-free bottles used for infant formula, hence considered safe and associated with positive sentiment.

In the NLTK regression results, infant cereals are associated with positive sentiment. It is noticeable that “pesticides” are prevalent among discussion of infant cereals, as well as “preservatives, sweeteners and additives”. Both hazard categories are themselves associated with a positive sentiment (although not 5% significant), which can be justified by strict regulation preventing infant food from these substances. This may explain infant cereals being associated with positive sentiment.

The *Campylobacter* and *Cronobacter* cases illustrate well the role of subjectivity and modality analyses in the understanding of concerns expressed by parents. In the case of *Campylobacter*, the language used is objective, which means the way the topic is addressed is not emotional but rather factual information and scientific evidence oriented. In the case of *Cronobacter*, the tone used is unconfident, which suggests that enhanced information and communication toward parents would be relevant on this topic.

## 5. Conclusion

The mobilization of the web analysis method presented, exploiting sentiment metrics, revealed relevant to bring answers about parents' perceptions of safety hazards in infant food products. From an application viewpoint, the results obtained regarding bisphenol A were somewhat unexpected, yet observed in both methods used. From a methodological viewpoint, the combination of analyses used allowed providing an explanation for these unexpected results, which was hardly possible in earlier questionnaire-based approaches. Another plus-value of the study is its status of first attempt in the domain explored, thus allowing initiating new ontologies for the explored field.

A possible limitation of the study lies in its format—online forums—which might bring a bias in the representativeness of the population surveyed. The results will thus need to be confirmed through complementary strategies. On-going work to collect perceptions from a larger geographic area will further identify common and divergent phenomena in various European countries.

A practical implication of these results concerns infant formula, which is the food product that raises the most concerns. Hence, special attention has to be paid to communication regarding this food product, whose use is recommended by reference authorities. Confirmation of these results should also lead to policy recommendations in order to (i) take into account users' priority concerns in risk management, and (ii) increase most users' awareness of safety hazards.

## Data availability statement

The raw data supporting the conclusions of this article will be made available by the authors, without undue reservation.

## Author contributions

RT, GH, and YP contributed to conception and design of the study, provided methodological resources, and supervised and administrated the work. SA collected and prepared the data and performed the analysis. RT and SA wrote the first draft of the manuscript. All authors contributed to manuscript revision, read, and approved the submitted version.

## References

[B1] Aceves Lara C. A. Athès V. Buche P. Della Valle G. Farines V. Fonseca F.. (2018). The virtual food system: Innovative models and experiential feedback in technologies for winemaking, the cereals chain, food packaging and eco-designed starter production. Innov. Food Sci. Emerg. 46, 54–64. 10.1016/j.ifset.2017.10.006

[B2] Aline S. (2021). Evaluating Consumer Health Concerns in Online Parenting Communities. Technical Report, Master thesis. France: Toulouse School of Economics.

[B3] ANSES. (2016). Dossier de presse, l'anses présente les résultats de son étude sur les expositions alimentaires aux substances chimiques des enfants de moins de trois ans. Available online at: https://www.anses.fr/fr/system/files/PRES2016DPA09.pdf (accessed July, 2021).

[B4] Bird S. Klein E. Loper E. (2009). Natural Language Processing with Python: Analyzing Text with the Natural Language Toolkit. Sebastopol, CA: O'Reilly Media, Inc.

[B5] Craven B. Islam S. M. (2011). “Ordinary least-squares regression,” The SAGE Dictionary of Quantitative Management Research (Thousand Oaks, CA: SAGE Publications). p. 224–228.

[B6] De Smedt T. Daelemans W. (2012). Pattern for python. J. Mach. Learn Res. 13, 2063–2067.

[B7] Djekic I. Mujcinović A. Nikolic A. Jambrak A. R. Papademas P. Feyissa A. H.. (2019). Cross-european initial survey on the use of mathematical models in food industry. J. Food Eng. 261, 109–116. 10.1016/j.jfoodeng.2019.06.007

[B8] European Commission (2021). Food for Infants and Young Children. Available online at: https://ec.europa.eu/food/safety/labelling-and-nutrition/specific-groups/food-infants-and-young-children_en (accessed February 08, 2023).

[B9] Franc-Dabrowska J. Ozimek I. Pomianek I. Rakowska J. (2021). Young consumers' perception of food safety and their trust in official food control agencies. Br. Food J. 123, 8. 10.1108/BFJ-11-2020-0992

[B10] Hulin M. Sirot V. Jean J. Héral V. Traore T. Mahé A.. (2019). Etude frande l'alimentation totale infantile: principaux résultats et recommandationsFrench infant total diet study: Main results and recommendations. Cahiers de Nutrition et de Diététique. 54, 275–285. 10.1016/j.cnd.2019.06.003

[B11] Hutto C. J. Gilbert E. (2014). “Vader: A parsimonious rule-based model for sentiment analysis of social media text,” in Proceedings of the International AAAI Conference on Web and Social Media. (Palo Alto, California: AAAI Press). 10.1609/icwsm.v8i1.14550

[B12] Kansou K. Laurier W. Charalambides M. N. Della-Valle G. Djekic I. Feyissa A. H.. (2022). Food modelling strategies and approaches for knowledge transfer. Trends Food Sci. Technol. 120, 363–373. 10.1016/j.tifs.2022.01.02130963919

[B13] Kim Y. Jeong S. R. (2015). Opinion-mining methodology for social media analytics. KSII T Internet Info. 9, 391–406. 10.3837/tiis.2015.01.024

[B14] Kurtz A. Thomopoulos R. (2021a). Consumer perceptions of infant food safety in France. Data INRAE Repository, V1. 10.15454/ZPPOJH

[B15] Kurtz A. Thomopoulos R. (2021b). Safety vs. sustainability concerns of infant food users: French results and european perspectives. Sustainability. 13, 18. 10.3390/su131810074

[B16] Mikolov T. Chen K. Corrado G. Dean J. (2013a). Efficient estimation of word representations in vector space. arXiv. 10.48550/arXiv.1301.3781

[B17] Mikolov T. Sutskever I. Chen K. Corrado G. S. Dean J. (2013b). Distributed representations of words and phrases and their compositionality. Adv. Neural Inf. Process. Syst. 26. Available online at: https://proceedings.neurips.cc/paper/2013/file/9aa42b31882ec039965f3c4923ce901b-Paper.pdf (accessed February 08, 2023).

[B18] Netmums (2021). Netmums Forum: Pregnancy, Parenting and Family Life Chat. Available online at: https://www.netmums.com/coffeehouse/ (accessed February 08, 2023).

[B19] SAFFI Project. (2020–2024). Safe Food for Infants in the EU and China. Available online at: https://www.saffi.eu (accessed February 08, 2023).

[B20] Sandjong-Sayon D. Cancalon M. Kurtz A. Meurillon M. Bourlieu-Lacanal C. Engel E.. (2022). Aliments infantiles : *Perceptions des utilisateurs et nouvelles questions de recherche*. Toulouse, France: Journées Francophones de Nutrition.

[B21] Stigler S. M. (1989). Francis galton's account of the invention of correlation. Statistical Sci. 4, 73–79. 10.1214/ss/1177012580

[B22] Tao D. Yang P. Feng H. (2020). Utilization of text mining as a big data analysis tool for food science and nutrition. Compr. Rev. Food Sci. Food Saf. 19, 875–894. 10.1111/1541-4337.12540 33325182

[B23] Thomopoulos R. Baudrit C. Boukhelifa N. Boutrou R. Buche P. Guichard E.. (2019). Multi-criteria reverse engineering for food: genesis and ongoing advances. Food Eng. Rev. 11, 44–60. 10.1007/s12393-018-9186-x

[B24] Vidal L. Ares G. Machín L. Jaeger S. R. (2015). Using twitter data for food-related consumer research: a case study on “what people say when tweeting about different eating situations”. Food Qual. Prefer. 45:58–69. 10.1016/j.foodqual.2015.05.006

[B25] Wankhade M. Rao A. C. S. Kulkarni C. (2022). A survey on sentiment analysis methods, applications, and challenges. Artif. Intell. Rev. 55, 5731–5780. 10.1007/s10462-022-10144-132950594

